# Bacterial and fungal pathogens causing neonatal sepsis and associated antimicrobial resistance in South African neonatal units—a systematic review

**DOI:** 10.1093/jacamr/dlaf214

**Published:** 2025-12-02

**Authors:** Vindana Chibabhai, Kessendri Reddy, Angela Dramowski, Clarence Yah, Daynia Ballot, Nelesh Govender

**Affiliations:** Centre for Healthcare Associated Infections, Antimicrobial Resistance and Mycoses (CHARM), National Institute for Communicable Diseases of the National Health Laboratory Service, Johannesburg, South Africa; Faculty of Health Sciences, University of the Witwatersrand, Johannesburg, Private Bag 3, WITS 2050, South Africa; Division of Medical Microbiology and Immunology, Department of Pathology, Faculty of Medicine and Health Sciences, Stellenbosch University, Cape Town 7500, South Africa; Faculty of Medicine and Health Sciences, Stellenbosch University, PO Box 241, Cape Town 8000, South Africa; Faculty of Health Sciences, University of the Witwatersrand, Johannesburg, Private Bag 3, WITS 2050, South Africa; Faculty of Health Sciences, University of the Witwatersrand, Johannesburg, Private Bag 3, WITS 2050, South Africa; Centre for Healthcare Associated Infections, Antimicrobial Resistance and Mycoses (CHARM), National Institute for Communicable Diseases of the National Health Laboratory Service, Johannesburg, South Africa; Faculty of Health Sciences, University of the Witwatersrand, Johannesburg, Private Bag 3, WITS 2050, South Africa; University of Cape Town Faculty of Health Sciences, Western Cape, South Africa; University of Exeter MRC Centre for Medical Mycology, Exeter, UK

## Abstract

**Background:**

Pathogens causing neonatal sepsis have developed resistance to antimicrobial treatment, resulting in the convergence of two public health issues; neonatal mortality and antimicrobial resistance. There are a few published studies presenting data from South Africa regarding neonatal sepsis pathogen and resistance profiles.

**Methods:**

We conducted a systematic review of bacterial and fungal neonatal sepsis pathogens and antimicrobial resistance profiles from 2005 to 2022.

**Results:**

Nine studies were included from 1235 screened. Most studies were from two provinces in South Africa and were conducted at academic hospitals. A single study included data collected nationally. Significant heterogeneity was noted, precluding the value of conducting a formal meta-analysis. There was significant variability in prevalence of pathogens, dependent on whether studies included coagulase negative *Staphylococci* (CoNS) or not. Studies that included CoNs reported higher prevalence for Gram-positive organisms compared with Gram-negative organisms versus studies that did not include CoNS. A higher proportion of Gram negatives compared with Gram positives and fungi was noted. Consistently low susceptibility to WHO first line empiric therapy was reported in most studies and low susceptibility to second line therapy reported in some studies. Seven studies reported mortality, which ranged from 15.6% to 46.3%.

**Conclusion:**

The prevalence of pathogens causing neonatal sepsis in South Africa are consistent with those on the WHO list of priority bacterial and fungal pathogens. A high percentage resistance to WHO first and second line treatment is noted and emphasizes the importance of country specific surveillance for neonatal sepsis.

## Introduction

Sepsis, triggered by invasive bacterial and fungal infections, is a leading cause of neonatal mortality and long-term neurodevelopmental morbidity, particularly in low- and middle-income countries.^1^ The United Nations Child Mortality report 2023 highlights the concern that 64 countries worldwide, most of which are on the African continent, are at risk of missing the 2030 sustainable development goal of reducing neonatal mortality to ≤12 neonatal deaths per 1000 live births.^2^

Advances in healthcare and medical technology have improved survival of preterm infants. Consequently, neonates admitted to neonatal units are increasingly vulnerable to healthcare- associated infections.^3^ These infections are often caused by pathogens exhibiting substantial antimicrobial resistance (AMR), limiting treatment options.^4^ Antimicrobial resistance is a global problem, to which an estimated 1.2 million deaths were attributed in 2019, with almost 10 000 neonatal deaths per 100 000 attributed to bacterial AMR in Africa.^5^

The WHO empiric antibiotic recommendations for neonatal sepsis have not been updated since 2005, despite newer editions of the guideline being published in 2013 and 2024.^6^ The South African Standard Treatment Guidelines (STG) and Essential Medicines List (EML) 2023 echo the WHO recommendations for empiric management of neonatal sepsis. However, updates are overdue owing to increasing pathogen AMR and a 3-fold increased odds of mortality in neonates and children receiving discordant empiric antibiotic treatment for infections.^7,8^

There are a few published studies presenting data from South Africa on the prevalence of pathogens causing neonatal sepsis and their resistance profiles. Thus, the local management guideline (STG Hospital Level—Paediatrics) has been unable to provide accurate, data-driven empiric therapy recommendations for early-onset and healthcare-associated neonatal sepsis.

Given this data gap, we performed a systematic review to describe the profile of bacterial and fungal neonatal sepsis pathogens at South African neonatal units from 2005 to 2022, aiming to determine pathogen prevalence, AMR profiles and bloodstream infection-associated mortality rates.

## Methods

### PECO framework

For population, neonates were defined as infants aged 1–28 days old who were admitted to neonatal units in South Africa during the defined study period (2005–2022), the exposure of interest was an episode of culture confirmed sepsis caused by pathogens of interest as defined by the study search criteria and their associated resistance profiles. No control group was included in this study. There were several outcomes for this analysis. They include the proportion of patients with bacterial or fungal neonatal sepsis, the proportion of patients with resistance to commonly used antimicrobial regimens and the percentage overall mortality.

### Search strategy and selection criteria

Data were sought on the aetiology of bacterial and fungal pathogens causing neonatal sepsis in South African neonatal units and their antimicrobial susceptibility profiles. We searched published and grey literature (reported between 2005 and 2022) including PubMed, MEDLINE, Web of Science and Scopus, and university library guides (LibGuides) from the University of the Witwatersrand, University of Pretoria, University of Cape Town, Stellenbosch University, University of Kwazulu Natal, Walter Sisulu University and the University of Free State. Conference websites searched include the Federation of Infectious Diseases Societies of Southern Africa, World Society for Paediatric Infectious Diseases, United South African Neonatal Association and ESCMID. We also searched surveillance reports on the National Institute for Communicable Diseases website.^9^

Each database was searched in January 2023 using Medical Subject Headings terms related to patient population, clinical infectious syndrome, pathogen, antimicrobial susceptibility, study type and geographical location. Population terms include ‘neonate’, ‘neonatal’, ‘newborn’, ‘low birth weight infant’, ‘premature’, ‘preterm’ ‘very low birth weight’, ‘extremely low birth weight’, ‘term’, ‘infant ‘South Africa’, ‘neonatal unit’ and ‘neonatal ward’. Clinical syndrome terms used include ‘bloodstream infection’, ‘blood culture’, ‘bacteraemia’, ‘bacteremia’, ‘fungaemia’, ‘fungemia’, ‘candidaemia’, ‘candidemia’, ‘septicaemia’, ‘septicemia’, ‘fever’, ‘pyrexia’, ‘neonatal sepsis’, ‘neonatal infection’, ‘late onset sepsis’, ‘late onset infection’, ‘healthcare associated infection’, ‘nosocomial infection’ and ‘hospital-acquired infection’. Study type search terms used were ‘observational studies’, ‘retrospective studies’, ‘case-control studies’, ‘cohort studies’, ‘follow-up studies’, ‘longitudinal studies’, ‘prospective studies’, ‘cross sectional studies’ and ‘epidemiology’. Pathogen terms used were ‘bacteria’, ‘bacterial’, ‘fungi’, ‘fungal’, ‘Gram negative’, ‘Gram positive’, ‘Enterobacterales’, ‘Enterobacteriaceae’, ‘Acinetobacter baumannii’, ‘Pseudomonas aeruginosa’, ‘Staphylococcus aureus’, ‘Enterococccus’, ‘Streptococcus’, ‘Listeria’, ‘Streptococcus agalactiae’, ‘Escherichia coli’ and ‘Candida’. Antimicrobial terms used were ‘antimicrobial’, ‘antibacterial’, ‘antibiotic’, ‘antifungal’, ‘resistance’, ‘resistant’, ‘surveillance’, ‘drug resistance’ and ‘microbial resistance’. Articles and reports written in English were included. Electronic page by page searches and reference list checks were conducted.

Review articles, editorials, policy statements, guidelines, case-control studies and commentaries were excluded, as were studies reporting only on HIV, mycobacterial infections, fungal infections other than candidaemia, non-sterile site infections, viral infections, syphilis and congenital infections. Studies were excluded if they presented data aggregated with regions outside of South Africa or if they presented data aggregated with other age groups where neonatal data could not be extracted. Where studies reported on fewer than 10 isolates, they were excluded. Studies reporting on a single pathogen or an outbreak in a neonatal unit were also excluded.

A title and abstract search was performed independently by two reviewers. Both investigators extracted study design information, baseline population characteristics, exposure details, number of cases, mortality and antimicrobial susceptibility data from all included studies into a standardized evidence table. Both reviewers cross checked the other reviewer’s data for accuracy.

Where duplicate reports were found originating from the same study, data were analysed from the most complete data set and remaining duplicates removed from the database. Disagreements regarding inclusion or exclusion of studies were resolved by consensus of the study team. Organisms regarded as commensals using the United States CDC National Healthcare Safety Network commensal list were excluded from analysis.^10^ CoNS infections were included if two cultures within 48–72 hours of each other identified the same species or if the study indicated that signs of sepsis accompanied the positive culture.

The study protocol was registered on PROSPERO (CRD42022341565) on 14 July 2022.^11^

### Data analysis

Screening data were entered and sorted through using Microsoft Excel and JBI SUMARI.^12^ Title and abstract screening was followed by full text screening. Completeness of data reporting, quality assessment and assessment of bias was performed using the STROBE and STROBE-NI tool, producing a percentage score for each study based on the tool’s parameters (each component of the tool was weighted equally). This was followed by independent data extraction by both reviewers. Data extraction parameters can be found in the accompanying online supplementary documents (Table S1, available as Supplementary data at *JAC-AMR* Online). Where discrepancies were noted, these were resolved by both reviewers examining the data from the studies together and where necessary, with the study team. Studies were included for analysis if they included both pathogen and antimicrobial susceptibility results.

The proportional representation of individual pathogens was calculated for each study, as well as the distribution of isolates by category: Gram-positive bacterial, Gram-negative bacterial and fungal pathogen groups. Proportion of resistance was calculated for each antimicrobial/antimicrobial combination for the same three organism categories.

The PRISMA checklist for systematic reviews was used to report the findings of this study.

## Results

**Figure 1.**
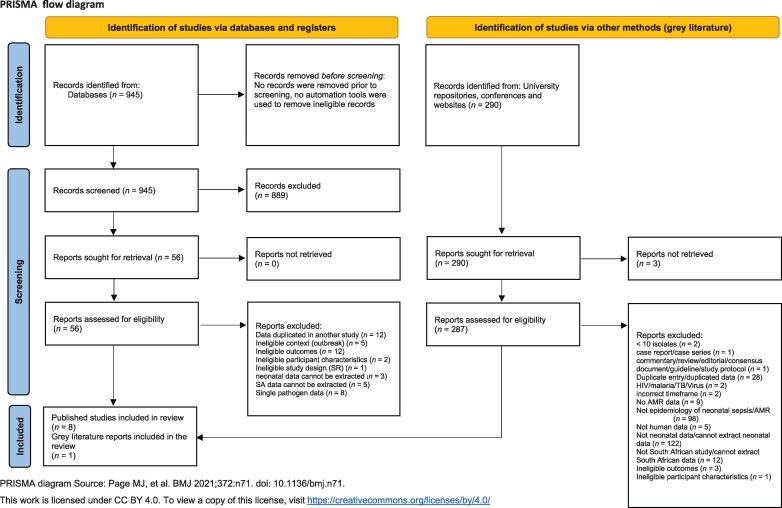
PRISMA of screening process.

The PRISMA chart in Figure 1 demonstrates the results of the search and selection process, including the reasons for exclusion. Nine studies were eligible for inclusion. A number of smaller studies were excluded as data overlapped with the study by Mashau *et al.*, which reported on isolates from sterile sites for all neonatal samples submitted nationally from the public health sector.^13^ At full text screening, eight publications were excluded as they reported on a single pathogen, often as a result of an outbreak.

The study characteristics of included studies is shown in Table 1. Most included studies were published between 2010 and 2019, with only a single study from 2005 to 2009. The largest included study was published in 2022.

**Table 1.** Study characteristics of included studies

Province	Year	Study design	Study site	study dates	Sample size	Specimen type	bacteria/ fungi	EOS/HAI	Reference
Gauteng	2013	cross-sectional study	Charlotte Maxeke Johannesburg Academic Hospital	Jan 2007—Dec 2011	59	blood culture	fungi	NR	Ballot^14^
Gauteng	2012	cross-sectional study	Charlotte Maxeke Johannesburg Academic Hospital	June 2009—June 2010	246	blood culture	bacteria	both	Ballot^15^
Western Cape	2015	cross-sectional study	Tygerberg Hospital	Jan 2009—Dec 2013	717	blood culture	both	HAI	Dramowski^16^
Gauteng	2017	cross-sectional study	Charlotte Maxeke Johannesburg Academic Hospital	Jan 2012—Dec 2012	236	blood culture	both	both	Lebea^17^
National	2022	cross-sectional study	National public sector hospitals	Jan 2014—Dec 2019	43 438	blood culture and CSF	both	both	Mashau^13^
Western Cape	2014	cross-sectional study	Tygerberg Hospital	Jan 2008—Dec 2008	78	blood culture	bacteria	not defined	Morkel^18^
Gauteng	2005	cross-sectional study	Chris Hani Baragwanath Hospital	July 2002—June 2003	135	blood culture	both	both	Motara^19^
Gauteng	2011	cross-sectional study	Chris Hani Baragwanath Hospital	Jan 2002—Dec 2004	112	blood culture	fungi	both	Nakwa^20^
Gauteng	2015	cross-sectional study	Chris Hani Baragwanath Hospital	March 2013—Sept 2014	228	blood culture	bacteria	EOS and CRBSI	Velaphi^21^

CRBSI, catheter associated bloodstream infection

Assessment of the completeness, quality and risk of bias was completed using the STROBE and STROBE-NI tool. Results of this assessment are shown in the Supplementary data (Table S2) with parameters numbered as described by Fitchett *et al.*^22^ Overall, studies met between 47% and 92% of the criteria listed in the STROBE and STROBE-NI tool. The study by Velaphi *et al.* obtained the highest quality score, while the lowest quality scores were noted for the studies by Lebea and Motara (47%).^17,19,21^

Although no meta-analysis was conducted due to heterogeneity in study design and outcome reporting, results were synthesized narratively. We summarized prevalence proportions across studies, reported resistance proportions where appropriate, and tabulated all-cause mortality for studies that reported this.

Summary statistics for prevalence of pathogens, AMR prevalence and mortality are shown in Tables 2–4. The most prevalent reported Gram-positive organisms varied per study. The studies by Motara *et al.* and Lebea *et al.* reported CoNS as the predominant Gram-positive organisms. *S. agalactiae* was the predominant Gram-positive organism reported in the study by Velaphi *et al.* and the study by Mashau *et al.* reported enterococci as the predominant Gram-positive organism. Among Gram negatives, there was more consistency with studies by Morkel *et al.*, Lebea and Mashau all reporting *K. pneumoniae* as the predominant Gram-negative organism, and only the study by Motara reporting *E.coli* as the predominant organism. There were differences in the *Candida* species reported with the studies by Nakwa and Ballot^14^ reporting a predominance of *C. parapsilosis* and the studies by Dramowski and Lebea reporting a predominance of *C. albicans*. Interestingly, the study by Mashau *et al.* reports similar proportions of *C. albicans* and *C. parapsilosis*.

**Table 2.** Proportion of pathogens reported per study

Reference	Nakwa *et al.*^20^	Motara *et al.*^19^	Ballot *et al.*^14^	Morkel *et al.*^18^	Dramowski *et al.*^16^	Ballot *et al.*^15^	Lebea *et al.*^17^	Velaphi *et al.*^21^	Mashau *et al.*^13^
	*n* (%)	*n* (%)	*n* (%)	*n* (%)	*n* (%)	*n* (%)	*n* (%)	*n* (%)	*n* (%)
Total isolates	N/A	135	N/A	78	796	246	236	104	43 438
Organism									
Gram-positive organisms									
Group B Streptococci	N/A	2 (1.5)	N/A	0	36 (4.5)	10 (4.1)	3 (1.3)	35 (33.7)	2495 (5.7)
*Listeria monocytogenes*	N/A	0	N/A	0	0	0	1 (0.4)	0	0
*Staphylococcus aureus*	N/A	3 (2.2)	N/A	15 (19.2)	112 (14.1)	23 (9.3)	33 (14.1)	3 (2.9)	5218 (12.0)
Enterococci	N/A	4 (3.0)	N/A	2 (2.6)	88 (11.1)	24 (9.8)	9 (3.8)	10 (9.6)	6579 (15.1)
CoNS	N/A	65 (48.1)	N/A	22 (28.2)	5 (0.6)	62 (25.2)	56 (23.7)	0	565 (1.3)
other	N/A	4 (3.0)	N/A	1 (1.3)	3 (0.4)	15 (6.1)	0	25 (24.0)	738 (1.7)
Gram-negative organisms									
*Klebsiella pneumoniae*	N/A	12 (8.9)	N/A	17 (21.8)	235 (29.5)	46 (18.7)	78 (33.1)	1 (1.0)	11 155 (25.7)
*Escherichiacoli*	N/A	20 (14.8)	N/A	2 (2.6)	58 (7.3)	23 (9.3)	14 (5.9)	10 (9.6	2496 (5.7)
*Enterobacter cloacae*	N/A	10 (7.4)	N/A	2 (2.6)	15 (1.9)	4 (1.6)	1 (0.4)	2 (1.9)	1319 (3.0)
*Serratia marcescens*	N/A	0	N/A	1 (1.3)	84 (10.6)	0	0	0	1446 (3.3)
*Acinetobacter baumannii*	N/A	0	N/A	14 (17.9)	69 (8.7)	27 (11.0)	20 (8.5)	3 (2.9)	5686 (13.1)
*Pseudomonas aeruginosa*	N/A	2 (1.5)	N/A	1 (1.3)	12 (1.5)	4 (1.6)	1 (0.4)	1 (1.0)	631 (1.5)
other	N/A	3 (2.2)	N/A	1 (1.3)	46 (5.8)	8 (3.3)	0	11 (10.6)	2103 (4.8)
Fungi									
*Candida albicans*	43 (38.4)	8 (5.9)	16 (27.1)	N/A	18 (2.3)	N/A	13 (5.5)	N/A	965 (2.2)
*Candida parapsilosis*	63 (56.3)	2 (1.5)	32 (54.2)	N/A	9 (1.1)	N/A	5 (2.1)	N/A	1014 (2.3)
*Candida auris*	0	0	0	N/A	0	N/A	0	N/A	60 (0.1)
*Candida glabrata*	4 (3.6)	0	2 (3.4)	N/A	2 (0.3)	N/A	2 (0.8)	N/A	0
Other	2 (1.8)	0	9 (15.3)	N/A	4 (0.5)	N/A	0	N/A	968 (2.2)

**Table 3.** Proportion susceptibility to various antimicrobial regimens

Reference	Nakwa *et al.*^20^	Motara *et al.*^19^	Ballot *et al.*^14^	Morkel *et al.*^18^	Dramowski *et al.*^16^	Ballot *et al.*^15^	Lebea *et al.*^17^	Velaphi *et al.*^21^	Mashau *et al.*^13^
	*n*/*N* (%)	*n*/*N* (%)	*n*/*N* (%)	*n*/*N* (%)	*n*/*N* (%)	*n*/*N* (%)	*n*/*N* (%)	*n*/*N* (%)	*n*/*N* (%)
total isolates	N/A	135	N/A	78	796	246	236	104	43 438
penicillin	N/A	N/A	N/A	3/3 (100.0)	N/A	N/A	N/A	34/34 (100.0)	4638/10 209 (45.4)
cloxacillin	N/A	30/67 (44.8)	N/A	10/15 (66.7)	38/112 (33.9)	7/23 (30.4)	2/33 (6.1)	3/3 (1 000)	2370/4193 (56.5)
gentamicin	N/A	N/A	N/A	N/A	N/A	N/A	N/A	43/46 (93.5)	2185/3918 (55.8)
vancomycin	N/A	N/A	N/A	26/30 (86.7)	88/88 (100.0)	134/134 (100.1)	N/A	N/A	12 677/12 995 (97.6)
linezolid	N/A	N/A	N/A	N/A	N/A	N/A	N/A	N/A	9527/9635 (98.9)
ampicillin + gentamicin	N/A	N/A	N/A	14/27 (51.9)	N/A	14/47 (29.8)	5/93 (5.4)	2/11 (18.2)	8184/23 130 (35.4)
third-generation cephalosporin	N/A	38/42 (90.5)	N/A	11/26 (42.3)	150/230 (65.2)	40/69 (58.0)	20/93 (21.5)	11/11 (1 000)	7942/23 220 (34.2)
piperacillin- tazobactam + amikacin	N/A	N/A	N/A	19/38 (50.0)	N/A	66/69 (95.7)	84/100 (84.0)	N/A	15 800/20 512 (77.0)
carbapenem	N/A	N/A	N/A	20/37 (54.1)	392/392 (100)	73/73 (100.0)	81/98 (82.7)	N/A	16 357/22 039 (74.2)
Azole	51/69 (73.9)	N/A	42/59 (71.2)	9/9 (1 000)	30/33 (90.9)	N/A	N/A	N/A	N/A
amphotericin B	64/69 (92.8)	N/A	N/A	N/A	33/33 (100.0)	N/A	N/A	N/A	N/A
echinocandin	N/A	N/A	N/A	N/A	33/33 (100.0)	N/A	N/A	N/A	N/A

**Table 4.** All-cause mortality reported in included studies

	Died	Number of patients assessed	Percentage (%)
Nakwa *et al.*^20^	27	111	24.3
Motara *et al.*^19^	21	96	21.9
Ballot *et al.*^14^	29	63	46.0
Morkel *et al.*^18^	25	54	46.3
Dramowski *et al.*^16^	112	717	15.6
Ballot *et al.*^15^	40	181	22.1
Lebea *et al.*^17^	N/A	N/A	N/A
Velaphi *et al.*^21^	17	97	17.5
Mashau *et al.*^13^	N/A	N/A	N/A

The proportion of isolates demonstrating susceptibility to various antimicrobials varied among the studies. Ampicillin + gentamicin susceptibility ranged from 5.4% to 51.9%, third-generation cephalosporin susceptibility ranged from 21.5% to 100%, piperacillin/tazobactam + amikacin susceptibility ranged from 50% to 95.7% and azole susceptibility ranged from 71.2% to 100%. Seven of the nine included studies reported mortality. This ranged from 17.5% to 46.3%.

We did not conduct a formal assessment of publication bias (e.g. funnel plots) as no meta-analysis was performed. However, we considered the potential for reporting bias during screening and synthesis. Of the 1235 records screened, 290 (23.5%) were from grey literature sources, including surveillance reports, theses and institutional datasets.

The overall certainty of the evidence for prevalence of pathogens and AMR proportions was considered low to moderate due to inconsistent reporting across studies, and limited data on specific organism–antibiotic combinations. Certainty in mortality outcomes was also considered low, mainly due to high heterogeneity and differences in definitions. All included studies were observational, in keeping with prevalence studies, and thus inherently at risk of bias, which further reduced confidence in the estimates.

## Discussion

This is the first systematic review of neonatal sepsis episodes in South African neonatal units, describing the pathogen prevalence, AMR profile and associated mortality rates. Systematic reviews pooling surveillance data provide valuable analyses on the prevalence of pathogens and local susceptibility profiles. This review highlights the importance of analysis of provincial and national data to inform empiric antimicrobial therapy guidelines.

The quality of included studies was moderate, lacking laboratory based details on study design including sampling strategy, laboratory methods used, antimicrobial standards used for interpretation e.g. European Committee on Antimicrobial Susceptibility Testing (EUCAST) versus Clinical and Laboratory Standards Institute (CLSI), limited details on study setting as part of the study design and sparse details in results. These may be as a result of general limitations from the health system as electronic medical records are not generally available in the South African public healthcare sector.

The pathogen distribution was variable in the included studies, probably as a result of the heterogeneity. It may also be the result of temporal changes in epidemiology, as our review spanned 22 years. Studies that considered CoNS as clinically significant pathogens tended to report Gram-positive organisms as the predominant pathogen causing neonatal sepsis. Group B Streptococci was only in one included study. *L. monocytogenes* was not a common pathogen although we note excluding a study reporting a national outbreak of *L. monocytogenes* as a result of contaminated meat products.^23^ The cross-sectional study by Mashau *et al.* include in our review also excluded cases of *L. monocytogenes* to avoid skewing of the data as a result of the protracted outbreak.

Studies that generally considered CoNs as contaminants noted *K. pneumoniae* to be the predominant pathogen. The role of CoNS as a significant cause of neonatal sepsis has not been clearly identified. This is reflected in how studies reported and interpreted the clinical significance of CoNS. Other studies focussed on neonatal sepsis globally and in Africa noted Gram-negative as the predominant pathogen, followed by Gram-positive organisms and last fungi.^4,24,25^

Among fungal pathogens, a high prevalence of *C. parapsilosis* was noted from studies originating from Gauteng province. This aligns with findings from previous national surveillance data from South Africa.^26^

There were high rates of resistance to antibiotics with a Gram-positive spectrum of activity in the included studies. Susceptibility of antibiotics with Gram-negative organism activity is concerning with only the older studies demonstrating third-generation cephalosporin susceptibility >80%. The susceptibility findings in the included studies are consistent with national bloodstream infection data demonstrated on the National Institute of Communicable Diseases Antimicrobial Resistance dashboard.^27^ We noted high carbapenem resistance in studies from the Gauteng province and the study by Mashau *et al.*, which reported national data. However, none of these studies commented on the mechanism of carbapenem resistance. Carbapenemase (CPE) enzymes are the main driver of carbapenem resistance in South Africa and globally, with OXA-48 and NDM enzymes predominating locally.^28^ Despite their clinical relevance, the specific types of carbapenemases are often not routinely reported in epidemiological or surveillance studies. This represents a missed opportunity, as newer treatment options such as ceftazidime–avibactam and aztreonam–avibactam are tailored to target specific CPE classes. Including CPE enzyme data in epidemiological studies is essential to support the development of treatment guidelines and to inform antimicrobial stewardship and national policy decisions.

A systematic review and meta-analysis on the prevalence of carbapenem-resistant Gram-negative bacteria among neonates in Africa showed an overall 30% prevalence of resistance to carbapenems.^29^ This was largely driven by *A. baumannii*, which demonstrated a pooled resistance prevalence to carbapenems of 45.9%, while *K. pneumoniae* demonstrated 23.8% and *E. coli* 12.3% resistance. Findings related to antifungal resistance, however, differ from those of our study. A systematic review of *Candida* antifungal drug resistance in Africa demonstrated differing species prevalence and varying resistance rates to azole antifungals, ranging from 0% to 70% in different countries.^30^

The first line empiric therapy recommended for neonatal sepsis by the WHO is ampicillin plus gentamicin.^31^ The studies included in this systematic review indicate very low susceptibility to this combination. Cefotaxime or ceftriaxone are listed as alternative options. Our data show similarly poor susceptibility rates to cefotaxime, probably due to the presence of extended spectrum beta- lactamase enzymes. The SA-EML also recommends ampicillin + gentamicin as first line therapy.^32^ As second line therapy, the SA-EML recommends use of piperacillin- tazobactam plus amikacin. The studies included here showed variable susceptibility to this combination, emphasizing the importance of unit specific data analysis to guide empiric therapy regimens.

The mortality rates reported in the included studies were generally higher than the 17.6% mortality reported in a systematic review and meta-analysis of neonatal sepsis incidence and mortality by Fleischmann and colleagues, which included studies published from 1979 to 2019.^33^ An observational study of neonatal sepsis in 11 countries (NeoOBS) found extensive variability in mortality ranging from 1.6% to 27.3% among participating sites. The NeoOBS study also found that mortality was higher with Gram-negative pathogens and fungi compared with Gram-positive organisms.^4^ A recent publication that analysed the clinical findings and outcomes of neonates admitted to lower-tier hospitals in South Africa found a crude in-hospital mortality of 25.5%, probably as a result of limited resources in these hospitals.^34^

Owing to the high rate of heterogeneity among the included studies, meta-analysis could not be performed. Possible reasons for heterogeneity include inconsistency in definitions used (early- and late-onset sepsis versus healthcare-associated sepsis) and populations from critical care versus lower care units. Our review spanned two decades during which clinical practice may have changed along with laboratory diagnostic tests and antimicrobial access. Due to limited detail in the laboratory methodology descriptions, it remains unclear whether variability in antimicrobial susceptibility interpretation—such as the classification of intermediate results as resistant—may have contributed to the observed heterogeneity across studies. Furthermore, study quality and sample size probably also contributed to heterogeneity. These sources of heterogeneity are expected in systematic reviews based on observational data across diverse settings and time periods, and they highlight the need for standardized reporting and improved surveillance systems.

Our protocol excluded studies reporting on outbreaks as this would have skewed results towards individual pathogens and/or resistance profiles. However, undetected or unreported outbreaks that occurred during the individual study periods may also have affected the epidemiology during these studies. In this regard, the importance of robust infection control measures to prevent outbreaks cannot be overstated.^35^

The inclusion of grey literature probably reduced the risk of publication bias to some extent. Nonetheless, there was variability in outcome reporting across studies. Some studies separated data as early-onset sepsis (EOS) and late-onset sepsis (LOS) or healthcare-associated infection (HAI) EOS and LOS/HAI, where others aggregated EOS and HAI/LOS. Some studies reported prevalence of pathogens separately for EOS and LOS/HAI but reported aggregated AST data. Some papers only reported percentages, not actual numbers. Reporting of AMR varied between the different studies, where some studies reported the resistance mechanism (e.g. ESBL), while others reported phenotypic resistance to the antimicrobial (e.g. resistance to third-generation cephalosporins). Reports that did not include AMR data were excluded. This inconsistency in reporting and subsequent exclusion of studies may have introduced selective reporting bias in our study.

We also note a number of limitations to our study. We did not contact individual authors to clarify data that was missing or if they held unpublished data. This would potentially have resulted in improved accuracy of data and a larger number of included studies. Although national and international conference abstracts were included, university research days were not included and we may thus have missed smaller research studies presented at these. It is possible that resistance patterns changed over the study period, but no formal trend analysis was performed as part of this study. High heterogeneity across studies, probably due to methodological and reporting variability, limits the comparability of findings. Last, the search was limited to studies published up to 2022, which may have excluded relevant recent evidence.

### Conclusion

Our findings demonstrate a high pooled prevalence of Gram-negative pathogens causing neonatal sepsis at neonatal units in South Africa. This is combined with high rates of resistance to antimicrobials with activity against Gram-negative pathogens. Of great concern is the high rates of resistance to WHO first and second line empiric therapies and the >20% resistance to carbapenems, echoing findings from numerous other studies that the WHO empiric therapy recommendations for neonatal sepsis are no longer appropriate.

These findings warrant changes to the empiric treatment guidelines for neonatal sepsis in South Africa. It adds further weight to the urgent call for antimicrobial clinical trials in neonates, such as the NeoSep and NANO trials that are currently underway, and underscores the importance of improved diagnostic and prevention strategies for neonatal sepsis.^36,37^ Furthermore, updated empiric treatment guidelines for neonatal sepsis optimized by accurate, accessible laboratory antibiograms must be prioritized for this vulnerable patient population.

## Supplementary Material

dlaf214_Supplementary_Data

## Supplementary data

Tables S1 and S2 are available as Supplementary data at *JAC-AMR* Online.
