# Supplementary material for: Bacterial and fungal pathogens causing neonatal sepsis and associated antimicrobial resistance in South African neonatal units—a systematic review
Source: JAC Antimicrob Resist. 2025 Dec 2;7(6):dlaf214. doi: 10.1093/jacamr/dlaf214 (PMC12669888; doi:10.1093/jacamr/dlaf214)

**Supplementary Table 1: Extraction parameters**

| **Author** |
| --- |
| **Title** |
| **Published year** |
| **study dates** |
| **specimen types reported** |
| **Bacteria/Fungi** |
| **outbreak/prevalence/other** |
| **EOS/LOS or HAI** |
| **study years** |
| **Study method** |
| **Defn EONS** |
| **CoNs definition** |
| **EOS and HAI aggregated** |
| **AST aggregated** |
| **empiric 1st line Rx** |
| **empiric 2nd line Rx** |
| **empiric antifungal Rx** |
| **mortality** |
| **EOS prevalence per pathogen n/N** |
| **GBS** |
| **L.monocytogenes** |
| **S.aureus** |
| **E.faecalis** |
| **E.faecium** |
| **S.pneumoniae** |
| **CoNs** |
| **other GPs** |
| **K.pneumoniae** |
| **E.coli** |
| **E.cloacae** |
| **S.marcescens** |
| **A.baumannii** |
| **P.aeruginosa** |
| **Other GNs** |
| **C.albicans** |
| **C.parapsilosis** |
| **C.auris** |
| **C.glabrata** |
| **Other Candida** |
| **EOS AMR per pathogen n/N** |
| **penicillin** |
| **gentamicin** |
| **cloxacillin** |
| **vancomycin** |
| **MRSA** |
| **VRSA** |
| **VRE** |
| **linezolid** |
| **ampicillin +/- gentamicin** |
| **3rd gen cephalosporin** |
| **piptaz + amik** |
| **imipenem** |
| **meropenem** |
| **ESBL** |
| **AmpC** |
| **CRE** |
| **XDR** |
| **CR non fermenter** |
| **azole resistant** |
| **ampho B resistant** |
| **echinocandin resistant** |
| **HAI/LOS prevalence per pathogen n/N** |
| **GBS (LOS)** |
| **CoNs** |
| **S.aureus** |
| **E.faecalis** |
| **E.faecium** |
| **Other GPs** |
| **K.pneumoniae** |
| **E.coli** |
| **E.cloacae** |
| **S.marcescens** |
| **A.baumannii** |
| **P.aeruginosa** |
| **Other GNs** |
| **C.albicans** |
| **C.parapsilosis** |
| **C.auris** |
| **C.glabrata** |
| **Other Candida** |
| **LOS/HAI AMR per pathogen n/N** |
| **Penicillin** |
| **gentamicin** |
| **cloxacillin** |
| **vancomycin** |
| **MRSA** |
| **VRSA** |
| **VRE** |
| **Linezolid** |
| **Ampi + genta** |
| **3rd gen ceph** |
| **piptaz + amik** |
| **imipenem** |
| **meropenem** |
| **ESBL** |
| **AmpC** |
| **CRE** |
| **MDR/XDR** |
| **CR - non fermenter** |
| **azole resistant** |
| **Ampho B resistant** |
| **Echinocandin resistant** |
| **Prevalence aggregated (EOS and LOS/HAI) combined n/N** |
| **GBS (LOS and EOS)** |
| **L.mono** |
| **CoNs** |
| **S.aureus** |
| **E.faecalis** |
| **E.faecium** |
| **Other GPs** |
| **K.pneumoniae** |
| **E.coli** |
| **E.cloacae** |
| **S.marcescens** |
| **A.baumannii** |
| **P.aeruginosa** |
| **Other GNs** |
| **C.albicans** |
| **C.parapsilosis** |
| **C.auris** |
| **C.glabrata** |
| **Other Candida** |
| **AMR aggregated (EOS and LOS/HAI) n/N** |
| **penicillin** |
| **gentamicin** |
| **cloxacillin** |
| **vancomycin** |
| **MRSA** |
| **VRSA** |
| **VRE** |
| **linezolid** |
| **ampicillin +/- gentamicin** |
| **3rd gen cephalosporin** |
| **piptaz + amik** |
| **imipenem** |
| **meropenem** |
| **ESBL** |
| **AmpC** |
| **CRE** |
| **MDR/XDR** |
| **CR non fermenter** |
| **azole resistant** |
| **ampho B resistant** |
| **echinocandin resistant** |

**Supplementary Table 2: Assessment of completeness, quality and risk of bias (STROBE and STROBE-NI)**


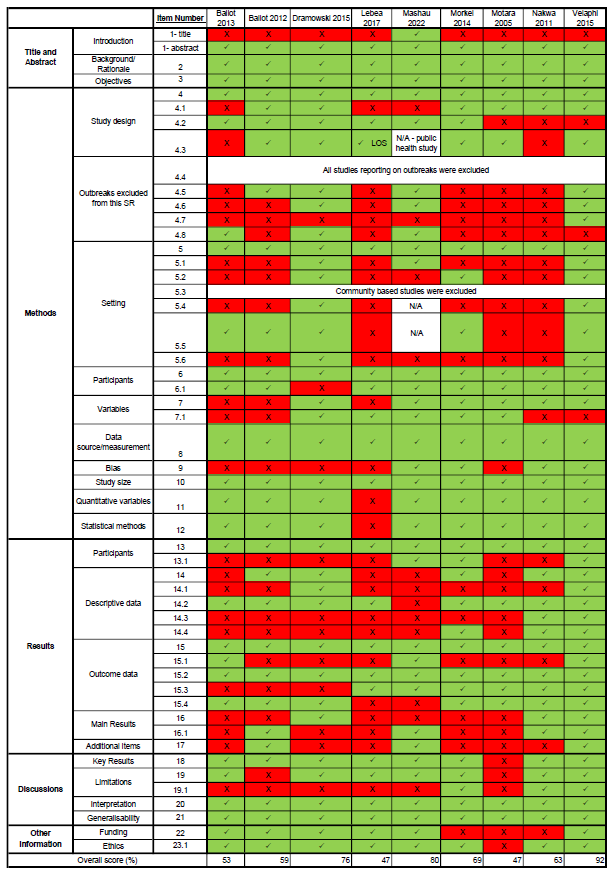

Supplement: dlaf214_Supplementary_Data [file dlaf214_supplementary_data.docx]
